# Ku proteins interact with activator protein-2 transcription factors and contribute to *ERBB2 *overexpression in breast cancer cell lines

**DOI:** 10.1186/bcr2450

**Published:** 2009-11-11

**Authors:** Grégory Nolens, Jean-Christophe Pignon, Benjamin Koopmansch, Benaïssa Elmoualij, Willy Zorzi, Edwin De Pauw, Rosita Winkler

**Affiliations:** 1 Laboratory of Molecular Oncology, GIGA Cancer, University of Liège, B34, avenue de l'hopital, Liege, 4000, Belgium; 2 Department of Human Histology-CRPP, University of Liège, B36, avenue de l'hopital, Liege, 4000, Belgium; 3 Laboratory of Mass Spectrometry; CART, GIGA, University of Liège, B6, avenue de la chimie, Liege, 4000, Belgium

## Abstract

**Introduction:**

Activator protein-2 (AP-2) α and AP-2γ transcription factors contribute to *ERBB2 *gene overexpression in breast cancer. In order to understand the mechanism by which the *ERBB2 *gene is overexpressed we searched for novel AP-2 interacting factors that contribute to its activity.

**Methods:**

Ku proteins were identified as AP-2α interacting proteins by glutathione serine transferase (GST)-pull down followed by mass spectrometry. Transfection of the cells with siRNA, expression vectors and reporter vectors as well as chromatin immunoprecipitation (ChIP) assay were used to ascertain the implication of Ku proteins on ERBB2 expression.

**Results:**

Nuclear proteins from BT-474 cells overexpressing AP-2α and AP-2γ were incubated with GST-AP2 or GST coated beads. Among the proteins retained specifically on GST-AP2 coated beads Ku70 and Ku80 proteins were identified by mass spectrometry. The contribution of Ku proteins to *ERBB2 *gene expression in BT-474 and SKBR3 cell lines was investigated by downregulating Ku proteins through the use of specific siRNAs. Depletion of Ku proteins led to downregulation of ERBB2 mRNA and protein levels. Furthermore, reduction of Ku80 in HCT116 cell line decreased the AP-2α activity on a reporter vector containing an AP-2 binding site linked to the *ERBB2 *core promoter, and transfection of Ku80 increased the activity of AP-2α on this promoter. Ku siRNAs also inhibited the activity of this reporter vector in BT-474 and SKBR3 cell lines and the activity of the *ERBB2 *promoter was further reduced by combining Ku siRNAs with AP-2α and AP-2γ siRNAs. ChIP experiments with chromatin extracted from wild type or AP-2α and AP-2γ or Ku70 siRNA transfected BT-474 cells demonstrated Ku70 recruitment to the *ERBB2 *proximal promoter in association with AP-2α and AP-2γ. Moreover, Ku70 siRNA like AP-2 siRNAs, greatly reduced PolII recruitment to the *ERBB2 *proximal promoter.

**Conclusions:**

Ku proteins in interaction with AP-2 (α and γ) contribute to increased ERBB2 mRNA and protein levels in breast cancer cells.

## Introduction

Breast cancer is the most common cancer in women in Europe [1]. Accumulation of different molecular alterations characterizes this complex disease. Five major breast cancer sub-groups have been distinguished according to gene expression signatures [2,3]. One of these subgroups is characterized by *ERBB2*/*Her2 *gene amplification and overexpression. This alteration is present in about 20% of breast cancers and was found to be predictive of poor prognosis before the development of *ERBB2 *targeted drugs [4-6].

The *ERBB2 *gene encodes for p185^-erbB2^, which is a transmembrane protein with intrinsic tyrosine kinase activity belonging to the EGF receptor (EGFR) family. No growth factor recognizing specifically *ERBB2 *with high affinity has been identified. Consequently, p185^-erbB2 ^is assumed to be activated by hetero-dimerization with another ligand-activated member of the EGFR family [6].

The high levels of p185^-erbB2 ^measured in breast cancer cells result from gene amplification and increased transcription rates [7,8]. In order to investigate the biology of these specific breast cancers, we chose to study the deregulation of *ERBB2 *gene expression. Analyses of the *ERBB2 *promoter have led to the identification of several regulatory sequences through which the gene is overexpressed. AP-2, Ets and YB-1 transcription factor families bind to some of these regulatory regions and have been shown to play a role in *ERBB2 *overexpression. Ets family transcription factors contribute to *ERBB2 *overexpression by binding to the proximal promoter [9]. YB-1 factors act through binding sites located 815 to 1129 bp upstream the main transcription initiation site [10], whereas AP-2 binding sequences (AP2BS) have been identified in the proximal [11-13] and distal [14] regions of the promoter.

The AP-2 transcription factor family contains five members: AP-2α, β, γ, δ and ε. All have a similar 50 kDa apparent molecular mass and are able to form homo- and hetero-dimers. They bind specific DNA sequences, AP2BS, through their conserved helix-span-helix DNA binding domain.

The involvement of AP-2α and AP-2γ factors in *ERBB2 *overexpression has been described in several breast cancer cell lines [11-13,15]. Besides the *ERBB2 *gene, AP-2 factors control the expression of several target genes implicated in the control of cell growth, differentiation and carcinogenesis [16].

AP-2 factors control transcription in association with transcriptional cofactors [17]. Among them, PC4, PARP [18], CITED-2, CITED-4 and CBP/p300 [19], as well as YY1 [20], have been shown to interact with and to contribute to AP-2 transcriptional activity. In our own research, we have observed a good correlation between p185^-erbB2^, AP-2α and YY1 expression levels in primary breast tumor samples [21]. Besides their role in transcription, cofactors are also important for the protection of AP-2 against proteasomal degradation [22].

In order to improve the current understanding of AP-2 (α and γ) activity, we sought here to identify further AP-2α interacting factors contributing to *ERBB2 *gene overexpression in breast cancer cells. We used a proteomic approach to isolate proteins interacting with this transcription factor in a BT-474 breast cancer cell line. Ku70 and Ku80 were identified by mass spectrometry among the AP-2α interacting proteins.

Ku 70 and Ku80 hetero-dimers are mostly known for their role, in association with DNA-PK, in the repair of DNA double strand breaks. However, it has been shown that Ku70 and Ku80 are involved in transcription regulation either by binding directly to DNA or through interaction with transcription factors [23]. Ku factors might also play a role in cancer [24].

We show that siRNAs targeting Ku mRNAs downregulate ERBB2 mRNA and protein levels. The use of reporter vectors containing the *ERBB2 *proximal promoter demonstrated that Ku70 and Ku80 proteins are involved in *ERBB2 *transcription regulation. Moreover, we show by ChIP assays that Ku70 protein is recruited to the *ERBB2 *gene promoter and its absence decreases AP-2α and AP-2γ recruitment. Furthermore, Ku70 recruitment is dependent on the expression of AP-2α and AP-2γ. These results contribute to a better understanding of the mechanism by which AP-2 factors upregulate *ERBB2 *gene expression in breast cancer cells.

## Materials and methods

### Cell lines

All the human cell lines (BT-474, ZR-75.1, MDA-MB-231, MCF-7 and SK-BR3, HepG2) were purchased from the American Tissue Culture Collection (Manassas, VA, USA). HCT116 and the derived 70/32 (Ku80+/-) cell lines were gifts from Dr. EA Hendrickson [25]. All the cells were cultured in the recommended media supplemented with 10% (v/v) fetal bovine serum, 2 mM glutamine and 100 μg/ml penicillin/streptomycin (Lonza, Basle, Switzerland).

### Antibodies

Mouse anti-AP-2α (3B5) [20], rabbit anti-AP-2α (C-18) [20], mouse AP-2γ (6E4/4) [21], goat anti-Ku70 (C-19) [26], goat anti-Ku80 (C-20) [26], mouse anti-Ku80 (B-1) [27], and control mouse, goat and rabbit IgG antibodies were purchased from Santa Cruz Biotechnology (Santa Cruz, CA, USA. Anti RNA Polymerase II (clone CTD4H8) and anti-β-actin (mAbcam 8226) antibodies were obtained from Abcam (Cambridge, UK).

### Plasmids and constructs

The pGEX2T-GST-AP2 and -GST vectors were gifts from Dr. Kannan [28]. The p86-AP2BS-Luc and p86-AP2BS mut-Luc plasmid reporter vectors (pGL3 basic reporter vector, Promega, Madison, WI, USA) have been previously described by Vernimmen et al [13]. The SV40-Luc control vector (pGL3 control vector) was purchased from Promega. The AP-2α and the corresponding control expression vectors [29] have been described previously [20]. Ku80 expression vector was a gift from Dr. Chen [30].

### GST-pull-down

GST-fusion proteins were expressed and purified according to the procedures provided by Amersham Bioscience (Buckinghamshire, UK). Their purification and the pull-down assay were carried out by using the MagneGST™ Pull-Down System (Promega, Madison, WI, USA). The manipulation (incubation and washing) of the beads was automated by the KingFisher robot (Thermo Fisher Scientific, Waltham, MA, USA). Washing buffer was phosphate buffer saline (PBS)/0,01% Tween. Procedure: 15 μl of MagneGST beads were washed twice with the washing buffer. Beads were incubated with 50 μl of sonicated *E. coli *(Bl21) transformed with plasmids encoding GST or GST-AP2alpha fusion protein in 250 μl of PBS for 30 min at room temperature (RT). After washing twice with 400 μl of washing buffer, beads bound with GST or GST-AP2 were incubated for 50 minutes with 100 μg (35 μl) of nuclear proteins extracted from BT474 at RT in 180 μl of PBS. After washing the beads three times with 400 μl, the interacting proteins were finally eluted by 8 M urea for 2D gel electrophoresis, or with Laemmli Buffer (2% SDS, 10% glycerol, 5% 2-mercaptoethanol, 0.125 M Tris HCl pH 6.8) for western blotting.

DNase I treatment - GST-AP2 beads incubated with the nuclear protein extracts were washed twice with PBS and suspended in DNase I buffer (40 mM Tris HCl, 10 mM NaCl, 6 mM MgCl2, 1mMCaCl2, pH 7.9). The suspension was incubated with increasing concentrations of DNase I (Roche, Basel, Switzerland) for one hour at 37°C. The suspension was washed three times with PBS, resuspended in Laemmli buffer and the bound proteins were eluted by vortexing during 10 min. Ku70, Ku80 and AP-2 were revealed by western blotting. DNA quantity was estimated by the picoGreen assay (Invitrogen, Carlsbad, CA, USA).

#### Two-dimensional gel electrophoresis and mass spectrometry

These techniques were performed as described previously [31], except that the proteins were directly loaded onto IPG strips (non linear IPG strip pH 4-10; Amersham, GE Europe (Buckinghamshire, UK). Liquid chromatography was carried out in an UltiMate™ pump/detection module, FAMOS™ micro autosampler, Switchos™ micro switching module (LC Packings, Dionex, Sunnyvale, CA, USA). The mass analysis was carried out in an ion trap Esquire HCT (Bruker Daltonics, Bremen, Germany) mass spectrometer. The database search was performed using a Mascot local server (Matrix Science, London, UK).

#### Immunoblotting

Proteins were separated on an SDS-PAGE (10%) and transferred to a PVDF membrane (Millipore, Billerica, MA, USA). Primary antibodies were used at a 1:1000 dilution. Secondary antibodies coupled with peroxydase (DAKO, Glostrup, Denmark) at a 1:4000 dilution were detected using the ECL system (Thermo Fisher Scientific).

**Immunoprecipitation **was carried out using Dynabeads Protein G (Invitrogen), according to the manufacturer's recommended protocol, using acetate sodium buffer for antibodies binding. Anti-AP-2α (C-18), Ku70 (C-19), Ku86 (C-20) antibodies and control antibody were used.

#### Transient transfection assays of reporter vectors

HCT116, 70/32 (HCT116 Ku80 +/-), BT-474 and SKBR3 cells were transfected using FuGENE HD reagent (Roche Applied Science). The cells (3 × 10^5^) were plated onto 24 mm tissue culture dishes, treated with FuGENE HD/DNA (ratio of 3:1) and incubated for 40 h in complete medium. Cells were then harvested. Lysis and enzymatic activity measures were carried out using the Luciferase Reporter Gene Assay kit (Roche Applied Science). Enzymatic activity was measured in a Wallac Victor™ luminometer (PerkinElmer, Waltham, MA, USA). The data were normalized to total protein content.

#### Transient siRNA transfection

siRNAs were transfected at a 30 nM final concentration using the Calcium Phosphate precipitation technique [32]. Cells were transfected twice at 48 h intervals. As a control, cells were transfected with the negative control siRNA OR-0030-neg05 (Eurogentec, Seraing, Belgium). The AP-2 siRNAs used were as previously described [14]. Other siRNAs were: Ku70-1, 5-GUGUGUACAUCAGUAAGAU; Ku70-2, 5-CAGGCAUCUUCCUUGACUU; Ku80-1, 5-GAAGAGGCAUAUUGAAAUA; Ku80-2, 5-CUCCAUUCCUGGUAUAGAA.

#### Reverse Transcription-Polymerase Chain Reaction (RT-PCR)

Total RNA was extracted after 90 hours of siRNA transfection treatment, using the High Pure RNA Isolation kit (Roche Applied Sciences). RNA quantification was carried out on a Nano Drop 1000 (Thermo Fisher Scientific). Reverse transcription was performed on 1 μg of total extracted RNA. Real time PCR analysis was performed on an ABI Prism 5700 apparatus (Applied Biosystems, Foster City, CA, USA) using the standard protocol. All the results were reported to the β-2-microglobulin mRNA quantity. The primers used were as previously described [21], except for Ku70: 5-AGAAGCAAACCGCCTGTA and 5-CAAGCCTCCTCCAATAAAGC; and Ku80: 5-TGCAGCAAGAGATGATGAGG and 5-GAAAGGCAGCTGCACATACA.

#### Chromatin Immunoprecipitation (ChIP)

Chromatin Immunoprecipitation was carried out using Dynabeads Protein G (Invitrogen). The previously described protocol [33] was adapted for higher chromatin quantities. On average for each immunoprecipitation reaction, 30 μg of chromatin DNA was precipitated with 4 μg of antibody. Rabbit anti-AP-2α (C-18), goat anti-Ku70 (C-19) and mouse anti-Ku80 (C-20) were used. Immunoprecipitation with pre-immune sera from the same animal species (Ig) or mock immunoprecipitation (NoAb) served as negative controls. Recovered DNA was quantified by real time PCR or by end-point PCR followed by agarose gel electrophoresis and the results were compared to known quantities of chromatin. The gene-specific primer sequences were: -6900 primers: 5-GCAGTAGCAAGCATCGAGTT and 5-TGGATCATCACAAAGGTTTTCA (-6981 bp to -6780 bp); -500-bp *ERBB2 *primers, 5-GACTGTCTCCTCCCAAATTT and 5-CTTAAACTTTCCTGGGGAGC (fragment -575 to -349 bp); -100-bp *ERBB2 *primers, 5-GCGAAGAGAGGGAGAAAGTG and 5-GGGGAATCTCAGCTTCACAA; GCK primers, 5-GGTAGAGCAGATCCTGGCAGAG and 5-TGAGCCTTCTGGGGTGGAGCGCA. Dilutions of known quantities of input DNA were used to quantify the PCR products.

## Results

### Identification of novel AP-2α interacting proteins

**Figure 1:**
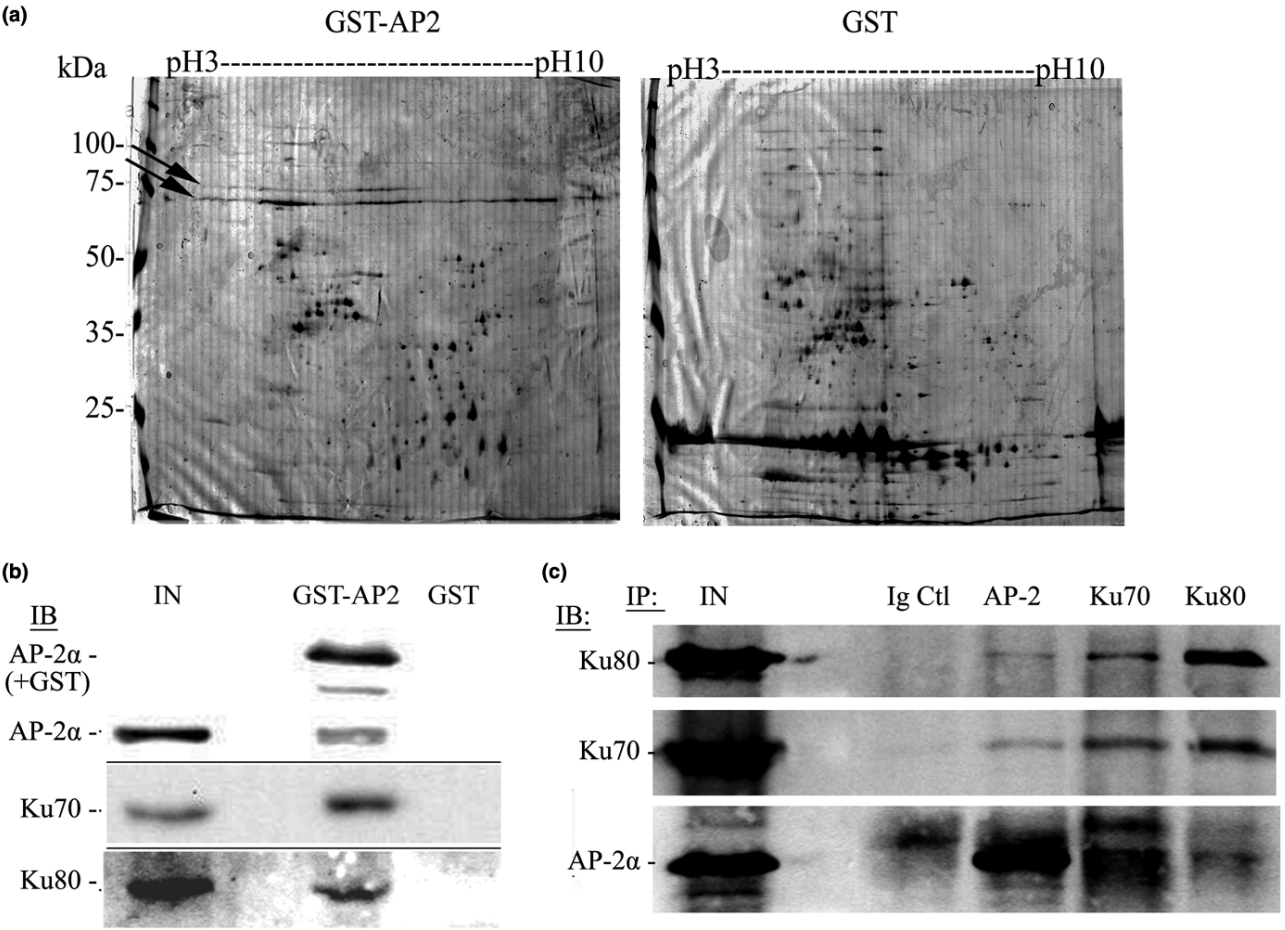
Identification of the Ku proteins interacting with AP-2α. **(a) **Two-dimensional gel electrophoresis of nuclear proteins eluted from GST AP-2α coated beads or beads coated with GST alone (GST). The gels were silver stained and the proteins retained on GST AP-2α beads were identified by mass spectrometry. Arrows show the location of Ku80 (up) and Ku70 (down). Molecular masses are indicated on the left and the pH scale on the top of the gels. **(b) **The presence of AP-2α and Ku proteins in the eluted fractions was verified by immunoblotting (IB) and compared to a fraction of the input (IN). **(c) **Co-immunoprecipitation (IP) of AP-2 and Ku proteins by AP-2, Ku70 and Ku80 antibodies or an Ig control (Ig Ctl). The proteins were revealed by immunoblotting (IB).

Our goal was to identify novel proteins interacting with AP-2, which contribute to the factor's transcriptional activity. Nuclear protein extracts from BT-474 breast cancer cells were incubated with a Glutathione-Serine-Transferase/AP-2α (GST-AP2) hybrid protein, linked to glutathione (GSH) coated magnetic beads. Beads coated with expressed GST alone were used as a negative control. After washes, the proteins bound to GST-AP2 or GST coated beads were eluted and resolved on two-dimensional gel electrophoresis. Two proteins migrating with an apparent molecular mass comprised between 70 and 100 kDa were repeatedly detected among the proteins eluted exclusively from the GST-AP2 coated beads (Figure 1A). The corresponding spots were cut out of the gel, the proteins digested, and the resulting peptides analyzed by LC-MS/MS. The proteins were identified as Ku70 and Ku80 [see Additional data file 1]. Most of the other spots on the 2D-gel recovered from the GST-AP2 pull down, were identified as GST or AP-2α fragments.

The presence of Ku proteins among the proteins eluted from the GST-AP2 beads was confirmed by immunoblotting with Ku specific antibodies (Figure 1B). Moreover, the interaction between AP-2 and Ku proteins was controlled by co-immunoprecipitation, using AP-2, Ku70 and Ku80 specific antibodies (Figure 1C). To verify that the binding of Ku proteins to AP2 is not due to the DNA contaminating the protein extracts [34], the mixture of GST-AP2 beads and nuclear proteins were incubated with increasing concentrations of DNase I. The result, [see Additional data file 2] showed a decrease in bound Ku proteins after treatment with low DNase I concentrations. However, the quantity of recovered Ku proteins remained constant when the enzyme concentration was raised, despite the steady decrease in contaminating DNA. This result indicates that the association of Ku to AP-2 is specific.

### Ku70/80 depletion induces downregulation of ERBB2 expression

**Figure 2:**
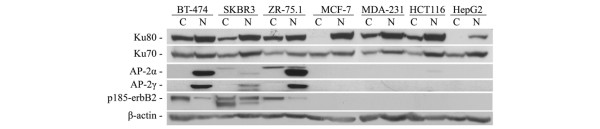
Ku70 and Ku80 protein levels in cytoplasm and nuclei of cancerous cell lines. 60 μg of nuclear and cytoplasmic proteins extracted from the cell lines were resolved by 10% PAGE. The Ku80, Ku70, AP-2α, AP-2γ and p185^erbB2 ^proteins were revealed by immunoblotting. The antibodies are indicated on left. Beta-actin (β-actin) protein was used as a loading control.

Figure 2 presents Ku70, Ku80 AP-2α, AP-2γ, and p185^-erbB2 ^protein levels in the cytoplasmic and the nuclear fractions of breast cancer (BT-474, SKBR3, ZR-75.1, MCF-7, MDA-MB-231), colon cancer (HCT-116) and hepatoma (HepG2) cell lines. In most cells, Ku70 and Ku80 proteins were detected in both the nuclear and the cytoplasmic fraction, with the exception of MCF-7 and HepG2 cell lines where Ku80 was exclusively nuclear. In comparison, AP-2α and AP-2γ factors were detected only in the nuclear fraction of BT-474, SKBR3 and ZR-75.1 breast cancer cells that overexpress p185^-erbB2 ^protein.

**Figure 3:**
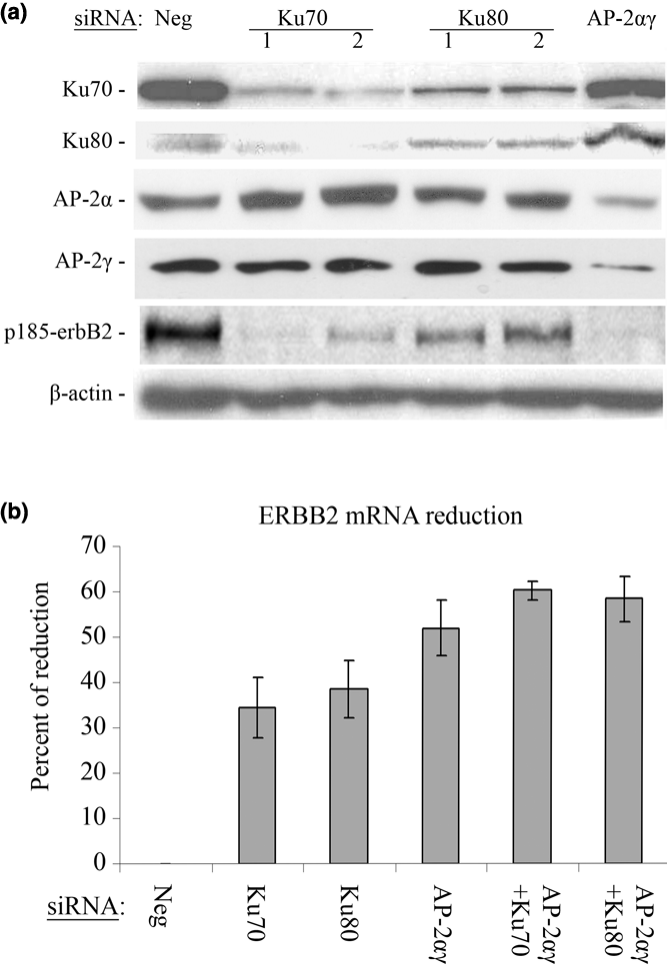
Effect of Ku70/Ku80 and AP-2 downregulation on ERBB2 protein levels in BT-474 cells. **(a) **Immunoblot showing p185^-erbB2^, Ku70, Ku80, AP-2α, AP-2γ and beta-actin (β-actin) protein levels in cells transfected with the siRNAs indicated above the photograph. **(b) **Percent reduction of ERBB2 mRNA levels compared to the Neg siRNA condition in cells transfected with the different siRNAs indicated below the figure. ERBB2 mRNA levels were quantified in triplicate by Real-time RT-PCR, and corrected for beta-2-microglobulin mRNA levels.

Next, we studied the consequence of Ku70 and Ku80 downregulation on the *ERBB2 *expression level. For that purpose, Ku70 and Ku80 were inhibited by the transfection of specific siRNAs in two *ERBB2 *overexpressing cell lines, BT-474 and SKBR3. In parallel, the cells were transfected with a combination of AP-2α and AP-2γ (AP-2αγ) siRNAs, previously shown to downregulate *ERBB2 *expression [21]. The levels of Ku70, Ku80, AP-2α, AP-2γ and p185^erbB2 ^proteins in BT-474 were assessed by western-immunobloting 96 hours after transfection (Figure 3A). Each siRNA inhibited its own target protein. Moreover, Ku70 and Ku80 siRNAs reduced both Ku70 and Ku80 protein levels, in agreement with published data [35,36].

The results of similar experiments on SKBR3 cells are presented in Additional data file 3, Figure A. Ku70 and Ku80 siRNAs reduced p185-^erbB2 ^levels less efficiently in SKBR3 than in BT-474 cells. We have to stress that AP-2αγ siRNAs were also less efficient in SKBR3 than in BT-474 cells.

In order to gain a more precise view of the cooperation between Ku and AP-2 proteins on *ERBB2 *gene transcription, the ERBB2 mRNA level was precisely quantified by real-time RT-PCR in Ku and AP-2αγ siRNA transfected cells. The ERBB2 transcript levels in cells transfected with the siRNAs were compared to the mRNA levels in cells transfected with the Negative (Neg) siRNA [Figure 3B and Figure B in Additional data file 3]. ERBB2 transcript levels were similarly reduced by Ku70 and Ku80 siRNAs in BT474 cells. The AP-2αγ siRNAs were more effective, reducing the ERBB2 transcript level by about 50%. Co-transfection of Ku and AP-2αγ siRNAs did not reduce further the ERBB2 mRNA level in these cells. While the Ku70 siRNA was as effective in SKBR3 as in BT-474 cells, Ku80 and AP-2 αγ siRNAs were much less effective. In contrast with BT-474 cells, in SKBR3 cells a combined effect of Ku70 and AP-2 siRNAs was observed.

### Ku70/80 regulate ERBB2 promoter transcriptional activity

**Figure 4:**
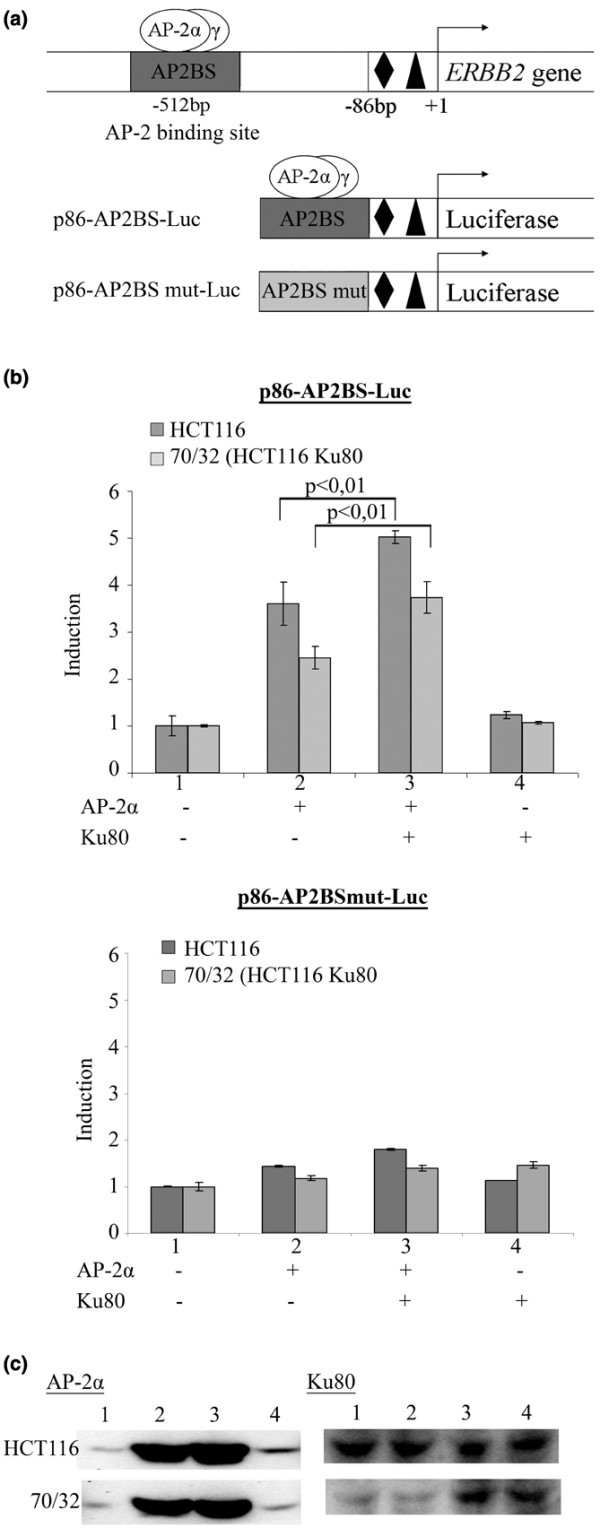
Influence of Ku80 on AP-2α transcriptional activity on *ERBB2 *gene promoter. **(a) **Schematic representation of the reporter vectors used in this study. All the reporter vectors contain 86 bp of the *ERBB2 *core promoter. The black diamond indicates the CAAT box; the black triangle the TATA box. The sequence of the AP2BS is that of AP-2 binding sites located at -512 bp from the main *ERBB2 *transcription start site. Mutation of three nucleotides abrogates the binding of the transcription factor to AP2BSmut [25]. **(b) **Variation of luciferase activity in cells transfected with (+) AP-α expression vector alone or with the Ku80 expression vector, in HCT116 and in 70/32 (HCT116 Ku80+/-) cell lines; compared with the activity in the cells transfected with the corresponding empty expression vectors (-). **(c) **Western blot showing the levels of AP-2α and Ku80 proteins in the cells transfected with the corresponding expression vectors.

To investigate the transcriptional control of *ERBB2 *gene expression by Ku and AP-2 proteins, we compared the HCT116 cell line and its derived 70/32 cell line, knocked out for one Ku80 allele [25]. Ku80 protein level in 70/32 cells was decreased by about 40-60% in comparison with the wild-type HCT116 cells (Figure 4C). The role of the interaction between Ku and AP-2 on ERBB2 gene expression was studied by transfecting in both cell lines a luciferase- reporter vector containing a wild type or a mutant AP-2 binding site (AP2BS) (Figure 4A). The cells were cotransfected with AP-2α and/or Ku80 expression vectors or the corresponding empty vectors. The luciferase activity in the cells transfected with the empty expression vector was considered as equal to one. As previously shown [13,20], AP-2α stimulated the activity of the reporter containing the wild type AP2BS (Figure 4B). However, the activation was reduced in 70/32 cells expressing less Ku80. Ku80 expression vector alone had no effect on the promoter activity. However, co-transfection of Ku80 with AP-2α expression vectors induced a significant increase in the promoter activity in both cell lines. Interestingly, transfection of Ku80 restored the activation capacity of AP-2α in 70/32 cell line. Figure 4C presents the AP-2α and Ku80 protein levels in the transfected cells.

**Figure 5:**
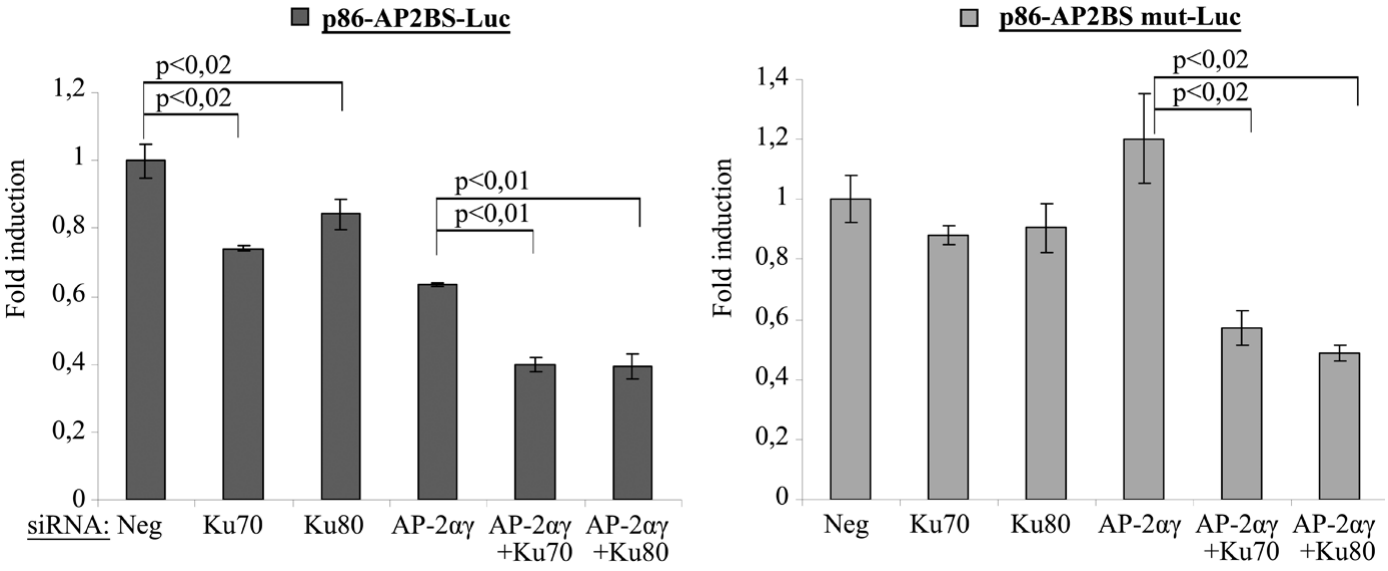
Ku70/80 proteins control *ERBB2 *promoter activity in BT-474 cell line.  Relative luciferase activity in cells transfected with the reporter vectors indicated above the figure, 72 hours after transfection of the siRNAs shown under each bar. The fold induction is the ratio between the luciferase activities in the cells transfected with the siRNA of interest and the activity measured in cells transfected with siRNA Neg. Luciferase activity was normalized to total protein content.

To complete this investigation, the reporter vectors activities were measured in BT-474 (Figure 5) and SKBR3 [see Additional data file 4] cells 72 hours after transfection of Ku and AP-2 siRNAs (Figure 5). The Luciferase activity in cells transfected with the negative siRNA was considered as equal to one in each condition. As expected, downregulation of AP-2α and AP-2γ inhibited only the activity of the reporter containing the functional AP2BS (p86-AP2BS-Luc). Ku70 or Ku80 siRNAs also inhibited significantly the activity of this reporter vector. Furthermore, when Ku70 or Ku80 siRNAs were co-transfected with the AP-2αγ siRNAs, the activity of p86-AP2BS-Luc was further reduced. In contrast, AP-2αγ, Ku70 and Ku80 siRNAs did not modify significantly the activity of the vector containing the mutated AP2BS (p86-AP2BS mut-Luc). However, co-transfection of AP-2αγ with Ku70 or Ku80 significantly reduced the activity of the reporter containing the mutant AP2BS.

Together, these results suggest that Ku proteins regulate *ERBB2 *gene expression through the proximal promoter, by an AP-2 dependent mechanism.

### Ku70/80 proteins are recruited to the ERBB2 proximal promoter

**Figure 6:**
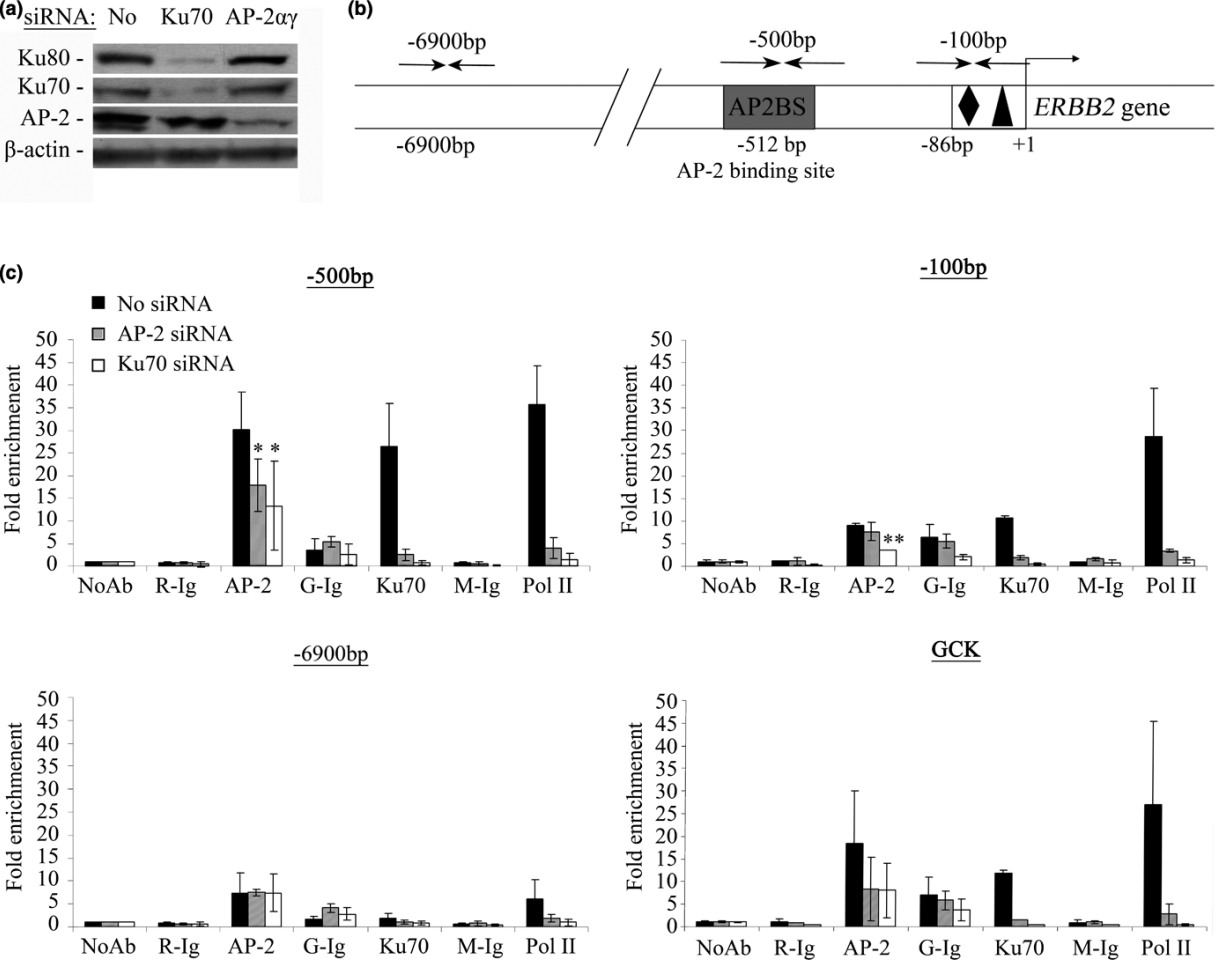
Ku70 is recruited to the *ERBB2 *promoter and is necessary for the recruitment of AP-2 and of RNA PolymeraseII.  **(a) **Chromatin extracted from BT-474 cells transfected or not with AP-2 (αγ) or Ku70 siRNAs (above) was shared by sonication to fragments of 100-500 bp. Protein expression was verified by immunoblot. **(b) **Schematic representation of the *ERBB2 *promoter illustrating the amplified regions. **(c) **DNA amplified after chromatin immunoprecipitation with AP-2, Ku70 and RNA Polymerase II (Pol II) specific antibodies. ChIP with control antibodies (Ig) are presented on the left of each experimental point (R: rabbit, G: goat and M: mouse). Graphs show the fold enrichment of the target sequence, compared to the no-antibody (NoAb) condition. The error bars were calculated from the results of three independent ChIP experiments. GCK sequence corresponds to a positive control for Ku recruitment. * and **: *P*-values < 0,05% compared to the No siRNA condition.

Next, we investigated Ku binding to *ERBB2 *gene promoter by chromatin immunoprecipitation (ChIP). Cross-linked chromatin was extracted from BT-474 (Figure 6A) and SKBR3 cells [Supplemental Data, Figure S.4]. We also extracted chromatin from the same cell type transfected with Ku or AP-2αγ siRNAs. Chromatin fragments were immunoprecipitated with antibodies recognizing AP-2α and AP-2γ (AP-2), Ku70, Ku80 and RNA Polymerase II (Pol II). The Pol II antibody was used as a positive control for the recruitment of the transcriptional machinery on the *ERBB2 *promoter [14,20,37]. Three regions of the *ERBB2 *promoter were amplified (Figure 6B). The -500 bp region contains a high affinity AP2BS [12]. The -100 bp region contains the CAAT and TATA boxes and corresponds to the *ERBB2 *core promoter. The -6900 bp sequence was used as an AP2BS negative control [14]. The Ku binding sequence from the Glucokinase (GCK) gene promoter was used as a positive control for Ku proteins binding [37]. Experiments were repeated three times and the average quantities of DNA were reported to the "No antibody" (NoAb) condition (Figure 6C).

In untreated BT-474 cells, Ku70 antibody immunoprecipitated most efficiently the -500 bp DNA sequence of the ERBB2 gene promoter (Figure 6C, black columns). In agreement with previously published data, AP-2 antibody also immunoprecipitated the same region [14,20]. Interestingly, AP-2 antibodies also immunoprecipitated the GCK sequence. The -500 bp, -100 bp and GCK sequences were efficiently recovered from Pol II specific immunoprecipitations. No DNA was recovered in the ChIP experiments using the C-20 Ku80 specific antibody (data not shown). Although the C-20 antibody has been successfully used in our immunoprecipitation experiments (Figure 1C) and published in ChIP data [26], we did not succeed to immunoprecipitate even the GCK control sequence with this antibody. As the -6900 bp region is devoid of an AP2BS, we consider this signal as background. Compared to BT-474 cells, immunoprecipitation with Ku70 and PolII antibodies of the *ERBB2 *and *GCK *promoter regions in SKBR3 was very poor [see Additional data file 5].

AP-2αγ siRNAs reduced significantly the amount of DNA corresponding to the 500 bp region recovered after immunoprecipitation with the AP-2 antibody. Interestingly, AP-2αγ siRNAs drastically reduced all DNA fragments recovered after ChIP using the Ku70 antibody in the BT-474 cell line. The recruitment of the DNA fragment from the GCK promoter after the Ku70 ChIP was also reduced. These siRNAs completely inhibited Pol II recruitment to the *ERBB2 *and *GCK *promoters. In the two cell lines, Ku70 siRNA reduced AP-2 recruitment to the *ERBB2 *and *GCK *gene promoters. This siRNA also completely inhibited the recruitment of Ku70 and PolII to the promoters we had investigated in the BT-474 cell line.

The ChIP results suggest that both Ku and AP-2 proteins are recruited on the *ERBB2 *proximal promoter in BT-474 cells. Moreover, the binding of both factors is necessary for the recruitment of transcription machinery on the *ERBB2 *gene promoter in BT-474 and SKBR3 cell lines.

## Discussion

The aim of this study was to identify novel proteins interacting with and contributing to AP-2 transcription factor activity. Using GST-pull-down coupled with two-dimensional gel electrophoresis and mass spectrometry, Ku70 and Ku80 proteins were identified as AP-2α interactors. AP-2/Ku interaction was confirmed by co-immunoprecipitation. We showed that downregulation of Ku proteins by siRNAs induced a strong reduction in ERBB2 mRNA and protein levels in BT-474 and SKBR3 cells. These siRNAs also inhibited the activity of reporter vectors containing the *ERBB2 *proximal promoter. ChIP experiments revealed that Ku70 proteins were recruited to the *ERBB2 *promoter. Interestingly, the inhibition of AP-2α and AP-2γ expression by siRNA strongly reduced Ku70 recruitment to the *ERBB2 *promoter. Ku70 siRNA reduced by half the recruitment of AP-2 factors to the -500 bp region of the *ERBB2 *promoter containing a high affinity AP2BS. More importantly, Ku70 siRNA downregulated PolII recruitment to the ERBB2 promoter. These results show that the Ku proteins are involved in *ERBB2 *gene expression regulation by AP-2 in breast cancer cells.

In addition to their role in the repair of DNA double strand breaks, Ku proteins have been shown to control other important cellular processes such as transcription and apoptosis. Ku proteins modulate transcription by several mechanisms and these properties seem to be gene and cell specific. For example, DNA binding of the Ku70/Ku80 heterodimer is responsible for the downregulation of glycoprotein glycophorin B in non erythroid cells [38]. In contrast, Ku binding to apolipoprotein C-IV promoter stimulates the expression of the gene [39]. Another study showed that interleukins -13/-4 induced expression of the lipooxygenase-1 gene is mediated by the binding of Ku dimmers to the promoter [40]. C-jun expression is also stimulated by Ku80 and possibly by Ku70 binding to gene promoter [41]. Ku proteins can also influence transcription by interaction with transcription factors or cofactors. So, Ku binding inhibits ESE1, an Ets family transcription factor, from binding to DNA and thus its transcriptional activity [42]. Ku proteins might also be involved in elongation [37] and transcription reinitialization [43].

Ku proteins were also shown to modulate gene expression by binding to PARP-1 and to YY1, which were shown to interact with and influence AP-2 transcription factor activity. Indeed, PARP-1 is necessary to preserve the transcriptional activity of overexpressed AP-2 transcription factors [18]. The Ku - PARP interaction has opposing effects on transcription. Ku proteins inhibit the transcriptional activity of β-catenin-TCF4 complex by interfering with PARP-1 binding [44]. In contrast, the Ku70/Ku80 dimmer - PARP-1 complex stimulates the expression of the S10019 gene [45]. Ku - YY1 interaction inhibits α myosin heavy-chain gene expression in the heart [46], contrary to the consequence of YY1 interaction with AP-2 on ERBB2 gene expression [20].

## Conclusions

In summary, the present study contributes to a better understanding of the regulation of *ERBB2 *gene expression in breast cancer cells, by demonstrating the implication of Ku proteins together with AP-2 transcription factors in the expression of the oncogene. Our results implicate Ku proteins in breast cancer by contributing to *ERBB2 *gene overexpression and thus the accumulation of excessive amounts of the receptor. Understanding the mechanisms of *ERBB2 *gene deregulation might help in the development of drugs that target further key elements responsible for this important deregulation. Among these, inhibiting Ku activity might be evaluated as an adjuvant for ERBB2 targeted therapy in breast cancer. However, more work is needed in models resembling the *in vivo *situation to confirm the implication of Ku proteins in the accumulation of excessive amounts of ERBB2 protein in cancerous cells.

## Abbreviations

AP-2: Activator Protein 2; AP2BS: AP-2 binding site; bp: base pairs; ChIP: chromatin immunoprecipitation; EGFR: EGF Receptor; GCK: Glucokinase; GSH: Glutathion; GST: Glutathione Serine Transferase; Luc: Luciferase; MS: Mass Spectrometry; PAGE: Poly-Acrylamide Gel Electrophoresis; PBS: Phosphate Buffer Saline; Pol II: Polymerase II; RT: Room Temperature; siRNA: small interfering RNA.

## Competing interests

The authors declare that they have no financial competing interests.

## Authors' contributions

GN carried out all the studies and drafted the manuscript. JCP and BK helped in the studies and interpretation of the results. BE, WZ and EDP participated in the study design, revised the manuscript and provided important intellectual support. RW conceived of the study, participated in its design, coordination and interpretation of the results and finalized the manuscript. All authors read and approved the final manuscript.

## Supplementary Material

Additional data file 1 Mass Spectometry identification of the Ku proteins interacting with AP-2α. Click here for file

Additional file 2 that Ku and AP-2 protein interaction is specific. Click here for file

Additional file 3 effect of Ku70/Ku80 and AP-2 deregulation on ERBB2 mRNA and protein expression in SKBR3 cells. Click here for file

Additional file 4 that Ku70/80 proteins control *ERBB2 *promoter activity in SKBR3 cell line. Click here for file

Additional file 5 that Ku proteins are necessary for the recruitment of AP-2 on the proximal *ERBB2 *gene promoter in SKBR3 cell line. Click here for file
