# Supplementary material for: Ku proteins interact with activator protein-2 transcription factors and contribute to ERBB2 overexpression in breast cancer cell lines
Source: Breast Cancer Res. 2009 Nov 11;11(6):R83. doi: 10.1186/bcr2450 (PMC2815545; doi:10.1186/bcr2450)
Supplement: Additional data file 1 — Mass Spectometry identification of the Ku proteins interacting with AP-2α. [file bcr2450-S1.pdf]

Additional data 1 : Mass Spectrometry identification of the Ku proteins interacting with AP-2α

**Accession number***	**Protein name**	**Mass†**	**Peptides matched‡**	**Total score§**
P13010	ATP-dependent DNA helicase II, 80 kDa subunit	83091	13	499
P12956	ATP-dependent DNA helicase II, 70 kDa subunit	69953	7	209

* Accession numbers of the identified proteins in the UniProtKB/Swiss-Prot data base.

† Predicted molecular masses of identified proteins.

‡ Numbers of peptides identified from the amino acid sequences of assigned proteins.

§ Total probability scores from the Mascot search for identified proteins. Scores were >39 (limit).
