# Supplementary material for: Ku proteins interact with activator protein-2 transcription factors and contribute to ERBB2 overexpression in breast cancer cell lines
Source: Breast Cancer Res. 2009 Nov 11;11(6):R83. doi: 10.1186/bcr2450 (PMC2815545; doi:10.1186/bcr2450)
Supplement: Additional file 2 — that Ku and AP-2 protein interaction is specific. [file bcr2450-S2.pdf]

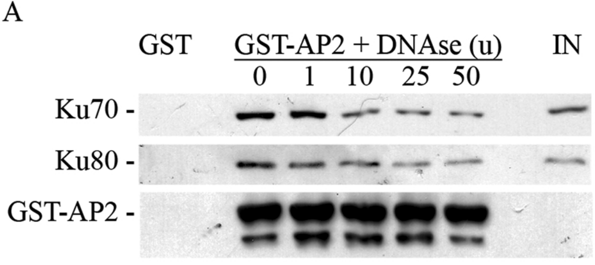

B

	DNA qty
GST	20,5ng
GST-AP2	
0 u	1122ng
1 u	934ng
10 u	307,5ng
25 u	194ng
50 u	69,5ng

Additional data 2 : AP-2 and Ku proteins interactions is specific

A. GST-AP-2 coated beads were incubated with nuclear protein protein extracts and increasing concentrations of DNAse I (Roche) at 37°C for 1 h. Bound proteins were eluted and resuspended in Laemmli buffer. Ku 70, Ku80 and AP2 proteins were revealed by western blotting in 25 µl of the eluates. Input (IN) was analyzed with 20 µg of protein nuclear extract of BT474 cells. B. DNA quantity (DNA qty) in the eluate was determined using picoGreen assay as described by the manufacturer (Invitrogen).
