# Supplementary material for: Ku proteins interact with activator protein-2 transcription factors and contribute to ERBB2 overexpression in breast cancer cell lines
Source: Breast Cancer Res. 2009 Nov 11;11(6):R83. doi: 10.1186/bcr2450 (PMC2815545; doi:10.1186/bcr2450)
Supplement: Additional file 3 — effect of Ku70/Ku80 and AP-2 deregulation on ERBB2 mRNA and protein expression in SKBR3 cells. [file bcr2450-S3.pdf]

A

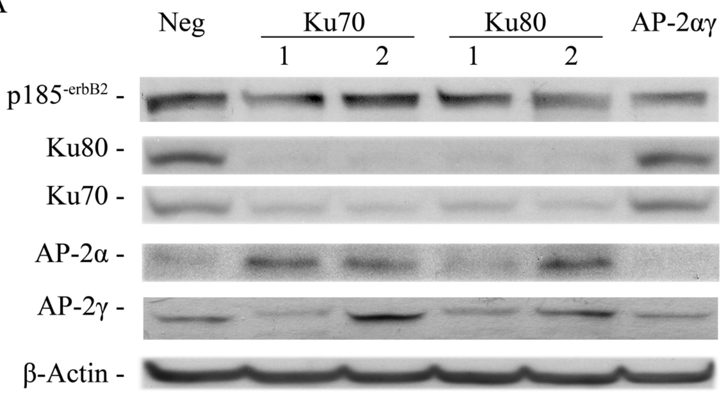

B

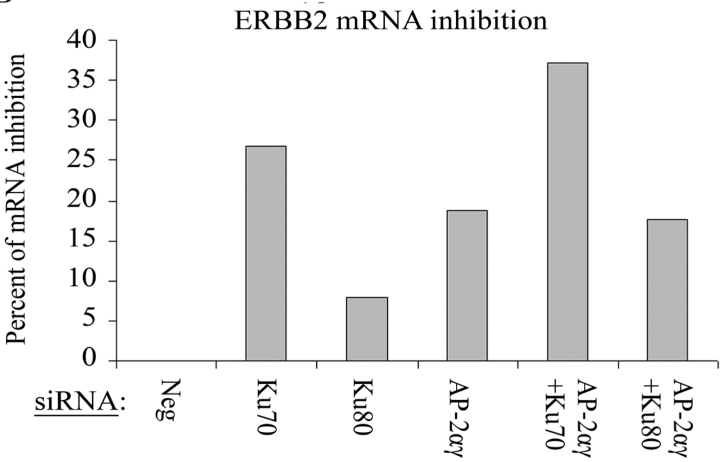

Additional data file 3 : Effect of Ku70 / Ku80 and AP-2 deregulation on ERBB2 mRNA and protein expression in SKBR3 cells.

A. Immunoblot analysis of p185erbB2, Ku70, Ku80, AP-2α, AP-2γ and beta-actin (β-Actin) protein expression from cells (SKBR3) transfected with two different Ku70 or Ku80 siRNA (see above), compared to the negative siRNA (Neg) and AP-2α and AP-2γ siRNAs (AP-2αγ). **B.** Real-time RT-PCR on the ERBB2 mRNA from SKBR3 cells transfected with different siRNAs (see down). Ku siRNA were combined with AP-2αγ or not. The graph represents the percentage of ERBB2 mRNA expression inhibition compared to the Neg siRNA condition. ERBB2 mRNA expression was corrected on the beta-2-microglobulin mRNA expression.
