# Supplementary material for: Ku proteins interact with activator protein-2 transcription factors and contribute to ERBB2 overexpression in breast cancer cell lines
Source: Breast Cancer Res. 2009 Nov 11;11(6):R83. doi: 10.1186/bcr2450 (PMC2815545; doi:10.1186/bcr2450)
Supplement: Additional file 4 — that Ku70/80 proteins control ERBB2 promoter activity in SKBR3 cell line. [file bcr2450-S4.pdf]

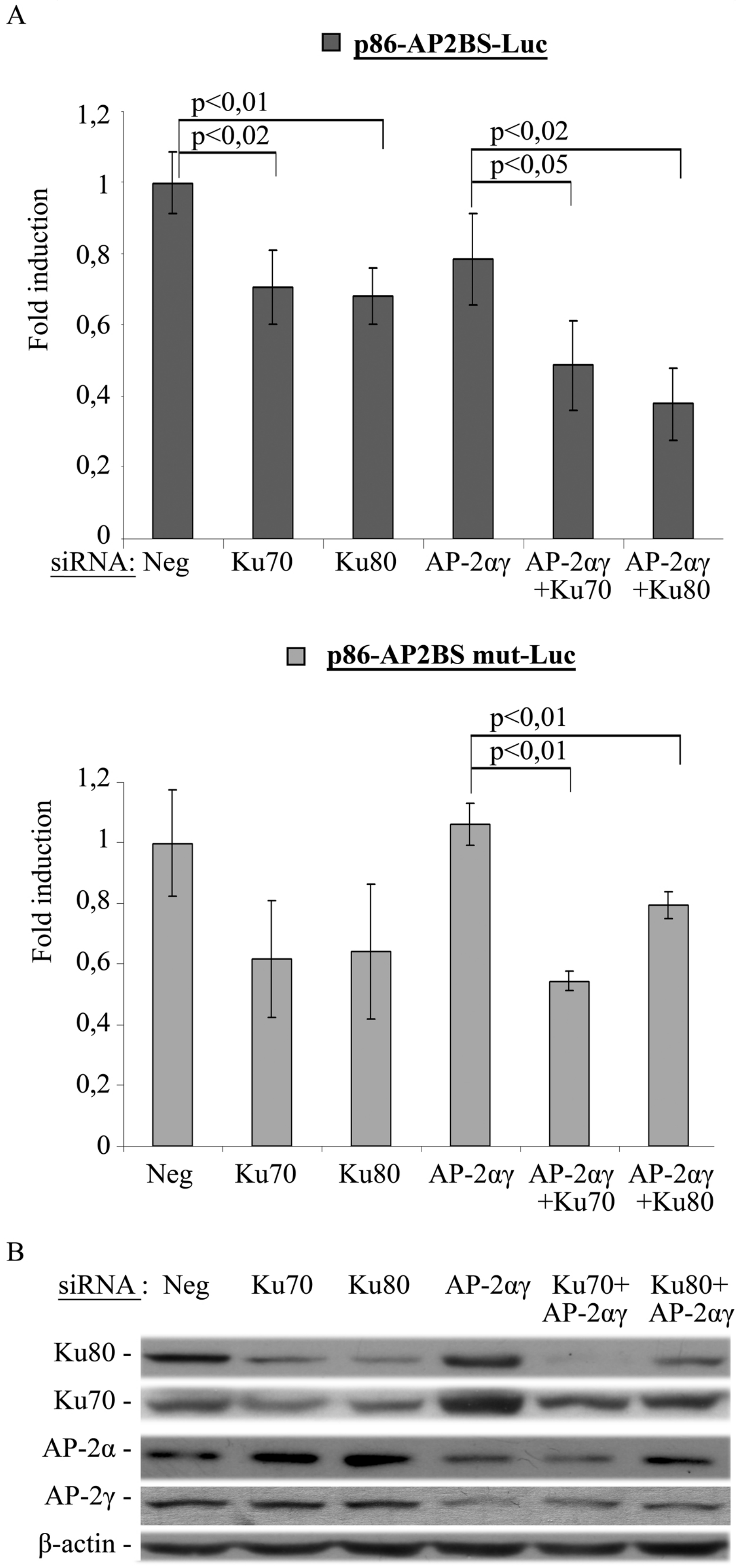

Additional data 4. Ku70/80 proteins control ERBB2 promoter activity in SKBR3 cell line.

A. Relative luciferase activity in cells transfected with the reporter vectors indicated above the figure, 72 hours after transfection of the siRNAs shown under each bar. The fold induction is the ratio between the luciferase activities in the cells transfected with the siRNA of interest and the activity measured in cells transfected with siRNA Neg. Luciferase activity was normalized to total protein content. **B.** Immunoblot control of the different siRNAs conditions from the experiment on point A. Antibodies are indicated on the left and siRNAs above the figure.
