# Supplementary material for: Ku proteins interact with activator protein-2 transcription factors and contribute to ERBB2 overexpression in breast cancer cell lines
Source: Breast Cancer Res. 2009 Nov 11;11(6):R83. doi: 10.1186/bcr2450 (PMC2815545; doi:10.1186/bcr2450)
Supplement: Additional file 5 — that Ku proteins are necessary for the recruitment of AP-2 on the proximal ERBB2 gene promoter in SKBR3 cell line. [file bcr2450-S5.pdf]

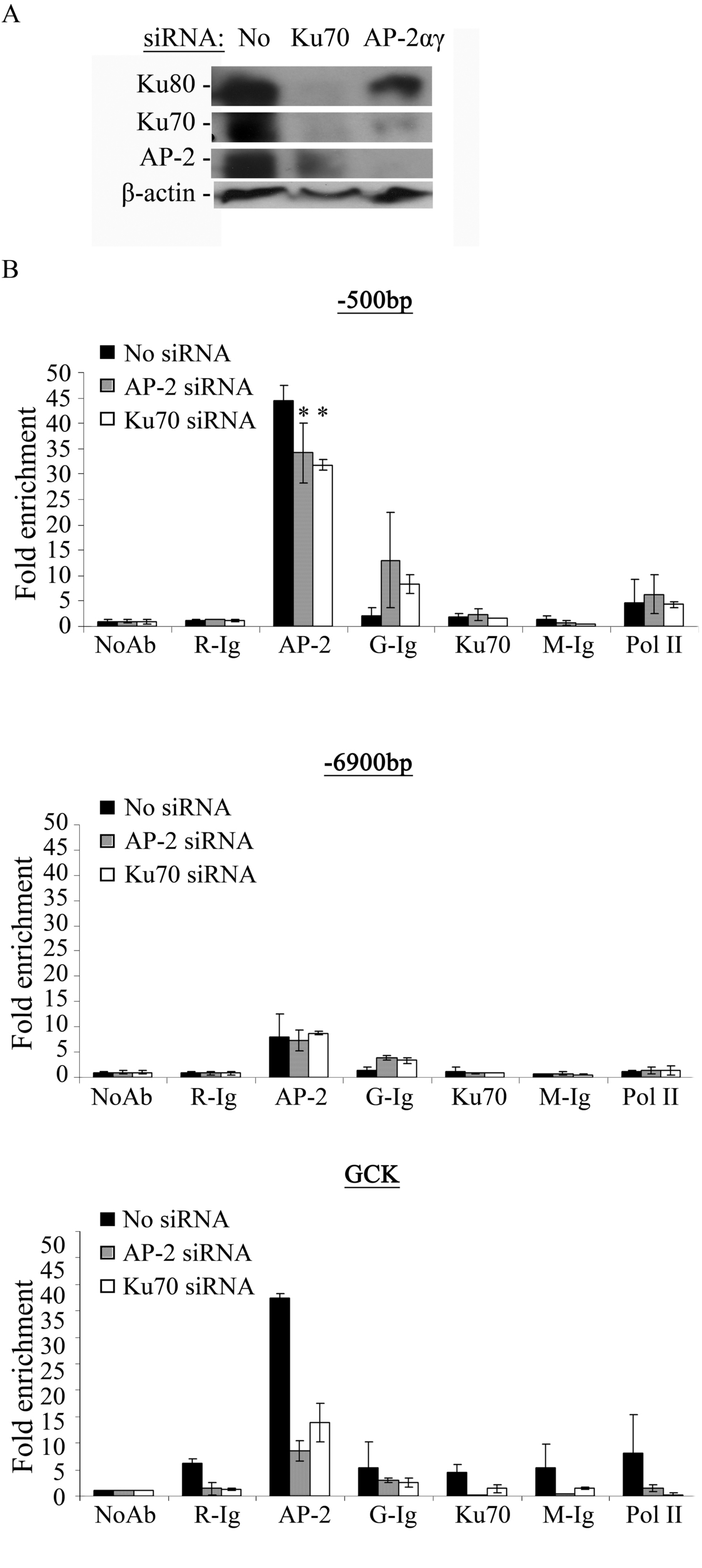

Additional data 5. Ku proteins are necessary for the recruitment of AP-2 on the proximal ERBB2 gene promoter in SKBR3 cell line.
A. Chromatin was extracted from SKBR3 cells transfected or not with AP-2 (αγ) or Ku70 siRNAs (above). Protein expression was verified by immunoblot. **B.** DNA amplified after chromatin immunoprecipitation with AP-2, Ku70 and RNA Polymerase II (Pol II) specific antibodies. ChIP with control antibodies (Ig) are presented on the left of each experimental point (R: rabbit, G: goat and M: mouse). Graphs show the fold enrichment of the target sequence, compared to the no-antibody (NoAb) condition. GCK sequence corresponds to a positive control for Ku recruitment. *: p-values <0,05% compared to the No siRNA condition.
